# Systemic Inflammation in Non-Demented Elderly Human Subjects: Brain Microstructure and Cognition

**DOI:** 10.1371/journal.pone.0073107

**Published:** 2013-08-26

**Authors:** Konstantinos Arfanakis, Debra A. Fleischman, Giorgia Grisot, Christopher M. Barth, Anna Varentsova, Martha C. Morris, Lisa L. Barnes, David A. Bennett

**Affiliations:** 1 Department of Biomedical Engineering, Illinois Institute of Technology, Chicago, Illinois, United States of America; 2 Rush Alzheimer's Disease Center, Rush University Medical Center, Chicago, Illinois, United States of America; 3 Department of Diagnostic Radiology and Nuclear Medicine, Rush University Medical Center, Chicago, Illinois, United States of America; 4 Department of Neurological Sciences, Rush University Medical Center, Chicago, Illinois, United States of America; 5 Department of Behavioral Sciences, Rush University Medical Center, Chicago, Illinois, United States of America; 6 Department of Internal Medicine, Rush University Medical Center, Chicago, Illinois, United States of America; Northwestern University Feinberg School of Medicine, United States of America

## Abstract

The purpose of this study was to test the hypothesis that higher levels of systemic inflammation in a community sample of non-demented subjects older than seventy years of age are associated with reduced diffusion anisotropy in brain white matter and lower cognition. Ninety-five older persons without dementia underwent detailed clinical and cognitive evaluation and magnetic resonance imaging, including diffusion tensor imaging. Systemic inflammation was assessed with a composite measure of commonly used circulating inflammatory markers (C-reactive protein and tumor necrosis factor-alpha). Tract-based spatial statistics analyses demonstrated that diffusion anisotropy in the body and isthmus of the corpus callosum was negatively correlated with the composite measure of systemic inflammation, controlling for demographic, clinical and radiologic factors. Visuospatial ability was negatively correlated with systemic inflammation, and diffusion anisotropy in the body and isthmus of the corpus callosum was shown to mediate this association. The findings of the present study suggest that higher levels of systemic inflammation may be associated with lower microstructural integrity in the corpus callosum of non-demented elderly individuals, and this may partially explain the finding of reduced higher-order visual cognition in aging.

## Introduction

Aging is linked to upregulation of inflammation-associated genes in the brain [1]–[3]. Inflammation has been associated with the pathogenesis and results of atherosclerosis [4], [5], response to ischemic tissue damage [6], the pathogenesis of Alzheimer's, Parkinson's and other neurodegenerative diseases [7]–[9]. Furthermore, inflammation in the elderly has been linked to worse cognitive function and increased rate of cognitive decline [10], [11], functional disability [12], frailty [13], and mortality [14]. Despite the important role of inflammation in aging, the relation between systemic inflammation and brain abnormalities in non-demented elderly human subjects has not been fully explored.

MRI has demonstrated macro-structural brain abnormalities linked to inflammation, prior to the onset of neurocognitive deficits and dementia. More specifically, it has been shown that, systemic inflammation in older persons without dementia is associated with reduced hippocampal and gray matter volume [15]–[17], as well as with cortical thinning [18], total brain atrophy [19], white matter hyperintense (WMH) lesions visible in T_2_-weighted MR images [4], [16], [20]–[22], and brain infarcts [22], [23]. Investigations of the microstructural integrity of brain tissue by means of diffusion tensor imaging (DTI) [24], [25] have demonstrated an association between systemic inflammation and reduced microstructural integrity in white matter pathways of non-demented individuals [26], [27]. However, previously published investigations of brain microstructure focused on middle-aged adults, or a combination of middle-aged and elderly adults mostly younger than 65 years of age. Due to advances in medicine, and due to social and environmental conditions, the number of people older than 65 years of age is expected to triple between 2000 and 2050, and the number of those older than 80 years of age is expected to quadruple (www.who.int). Therefore, further investigation is necessary into the relation between systemic inflammation and microstructural brain abnormalities in non-demented elderly subjects.

The purpose of this study was to test the hypothesis that, higher levels of systemic inflammation in a community sample of non-demented subjects older than 70 years of age are associated with reduced diffusion anisotropy in brain white matter and lower cognition. Older persons without dementia underwent detailed clinical and cognitive evaluation and MR imaging, including DTI. Systemic inflammation was assessed with a composite measure of commonly used circulating inflammatory markers (C-reactive protein, CRP, and tumor necrosis factor-alpha, TNFα) that have been linked to brain abnormalities in community-based samples of older persons [4], [19]. Voxel-wise analysis was used to investigate the link between systemic inflammation and diffusion measures in white matter. To minimize contamination of the results from any associations of inflammation with brain atrophy, infarcts, and WMHs, analysis was conducted on the skeleton of white matter and accounted for WMHs voxel-wise, using WMH masks generated for all subjects.

## Methods

### Ethics Statement

Human subjects were recruited from the Rush Memory and Aging Project, a longitudinal clinical-pathologic study of aging and Alzheimer's disease [28]. Recruitment for the Rush Memory and Aging Project began in September 1997 and is ongoing. All participants provided written informed consent. This study was approved by the Institutional Review Board of Rush University Medical Center, and was compliant with the Health Insurance Portability and Accountability Act.

### Participants

Participants agreed to annual testing and biannual brain MR imaging. Each participant underwent a uniform structured clinical evaluation, which included medical history, neurological examination, and cognitive testing [28]. Trained technicians administered cognitive tests for episodic memory (CERAD Word List Memory, Recall, and Recognition, immediate and delayed recall of the East Boston Story, Story A from Logical Memory), semantic memory (20-item version of the Boston Naming Test, 15-item version of Extended Range Vocabulary, 20-item reading recognition test from the National Adult Reading Test, Verbal Fluency), working memory (Digit Span Forward, Digit Span Backward and Digit Ordering), perceptual speed (oral version of the Symbol Digit Modalities Test, Stroop Test, Number Comparison), visuospatial ability (15-item version of Judgment of Line Orientation, 17-item version of Standard Progressive Matrices). Raw scores on each test were converted to z-scores and then averaged for each domain. A person's z-scores across all tests were averaged to yield a single composite score of global cognition [28]. This information was reviewed by a clinical neuropsychologist to diagnose cognitive impairment. Participants were then evaluated in person by an experienced clinician and were classified with respect to dementia and Alzheimer's disease in accordance with the criteria of the National Institute of Neurologic and Communicative Disorders and Stroke and the Alzheimer Disease and Related Disorders Association (NINCDS/ADRDA) [29], [30]. Diagnosis of dementia required a history of cognitive decline and evidence of impairment in at least 2 cognitive domains. Diagnosis of mild cognitive impairment (MCI) required diagnosis of cognitive impairment by the neuropsychologist without a diagnosis of dementia by the examining clinician. Individuals with dementia, brain surgery, brain tumors, contraindications for MRI, and those taking anti-inflammatory medications, were not included in this study. At the time of these analyses, 95 non-demented elderly individuals met eligibility requirements and produced images that passed quality control tests. The age of the participants was 85±6 years (range 73–100 years). Other demographic and clinical characteristics of the participants are presented in Table 1.

**Table 1:** Demographic and clinical characteristics.

Characteristics	
Total number of subjects, N	95
Age, y (SD)	85 (6)
Male, N (%)	26 (27%)
Education, y (SD)	15 (3)
C-reactive protein, µg/ml (min-max)	0.5 (0.009–4.1)
Tumor necrosis factor-alpha, pg/ml (min-max)	47 (19–224)
Body mass index, kg/m^2^ (SD)	27 (6)
History of diabetes, N (%)	16 (17%)
History of hypertension, N (%)	62 (65%)
History of smoking, N (%)	37 (39%)
Systolic blood pressure, mm Hg (SD)	133 (18)
Total cholesterol, mg/dl (SD)	177.2 (39)
High-density lipoprotein, mg/dl (SD)	61.1 (17)
Low-density lipoprotein, mg/dl (SD)	91.5 (31)
Using statin medication at evaluation, N (%)	52 (55%)
Using antihypertensive medication at evaluation, N (%)	64 (67%)
Using anticoagulants at evaluation, N (%)	20 (21%)
Episodic memory score (SD)	0.4 (0.6)
Semantic memory score (SD)	0.3 (0.6)
Working memory score (SD)	0.2 (0.7)
Perceptual speed score (SD)	0.1 (0.7)
Visuospatial ability score (SD)	0.4 (0.6)
Global cognition score (SD)	0.3 (0.5)

### Assessment of Systemic Inflammation

A standard procedure was used to collect blood samples. Plasma CRP and TNFα levels were quantified using highly sensitive multiplexed sandwich ELISA arrays designed to detect CRP, TNFα and other inflammatory biomarkers (Endogen Searchlight technologies, Billerica, MA). The lower detection limit for CRP was 0.6 pg/ml. Its intra-assay coefficient of variation was 6.8–7.0%, and its inter-assay coefficient of variation was 2.9–5.8%. The lower detection limit of TNFα was 2.3 pg/ml. Its intra-assay coefficient of variation was 3.1–5.0%, and its inter-assay coefficient of variation was 4.4–4.7%. The multiplex ELISA arrays were tested for spotting consistency and specificity to rule out the presence of cross-reactivity or nonspecific binding resulting from multiple antibody combinations. CRP values were adjusted by a correction factor based on data obtained from recalibration studies. Since high levels of two inflammatory markers likely constitute a more specific indicator of systemic inflammation than a high level of only one inflammatory marker [31], [32], a composite measure of inflammation was constructed as the sum of the z-scores of the log-transformed CRP and TNFα.

### Image Acquisition

Brain MR imaging was conducted on all participants using a 1.5 Tesla General Electric MRI scanner (Waukesha, WI). High-resolution T_1_-weighted anatomical data was obtained using a 3D magnetization-prepared rapid acquisition gradient-echo (MPRAGE) sequence with the following parameters: echo-time (TE) = 2.8 msec, repetition time (TR) = 6.3 msec, preparation time  = 1000 msec, flip-angle  = 8°, field-of-view (FOV) = 24 cm×24 cm, 160 sagittal slices, slice thickness  = 1 mm, no gap, 224×192 image matrix reconstructed to 256×256, and two repetitions. T_2_-weighted fluid attenuated inversion recovery (FLAIR) data was collected on all participants using a 2D fast spin-echo sequence with the following parameters: TE = 120 msec, TR = 8 sec, inversion time  = 2 sec, FOV = 24 cm×24 cm, 42 oblique axial slices, slice thickness  = 3 mm, no gap, 256×224 image matrix reconstructed to 256×256. Finally, spin-echo echo-planar DTI data was collected on all participants using the following parameters: TE = 84.6 msec, TR = 5.4 sec, FOV = 24 cm×24 cm, 36 oblique axial slices, slice thickness  = 3 mm, no gap, 128×128 image matrix reconstructed to 256×256, b = 900 sec/mm^2^ for 12 diffusion directions uniformly distributed in 3D space [33], two b = 0 sec/mm^2^ volumes, and 6 repetitions.

### Image Processing

For each participant, white matter lesions commonly present in the brain of elderly persons (often referred to as white matter hyperintensities, WMHs, due to their hyperintense appearance in T_2_-weighted images) were automatically segmented. To accomplish that, the T_1_-weighted MPRAGE data was first spatially registered to the T_2_-weighted FLAIR data using affine registration (FLIRT, FMRIB, University of Oxford, UK) [34]. The brain was extracted from the coregistered MPRAGE and FLAIR image volumes (BET, FMRIB, University of Oxford, UK) [35]. WMHs were then automatically segmented for each participant using a support vector machine classifier considering both T_1_-weighted MPRAGE and T_2_-weighted FLAIR information (WMLS, SBIA, University of Pennsylvania, PA) [36]. Maps of WMHs were generated for each participant (containing the value of one in WMHs and zero elsewhere).

The brain was extracted from the raw DTI data of each participant. Distortions caused by eddy-currents in the diffusion-weighted volumes, as well as bulk-motion, were corrected by 3D affine registration of all volumes to the first volume with no diffusion weighting (b = 0 sec/mm^2^). Distortions due to magnetic field non-uniformity and echo-planar imaging were corrected by non-linear registration to the corresponding T_1_-weighted MPRAGE data. The B-matrix was appropriately reoriented. The diffusion tensor in each voxel of the brain was then estimated using non-linear tensor fitting. Maps of the fractional anisotropy (FA), trace of the diffusion tensor, axial and radial diffusivity, were produced for each participant [24], [25], [37]. All DTI data pre-processing was accomplished using TORTOISE (http://www.tortoisedti.org) [38].

The WMH mask of each participant was converted to the space of the corresponding processed DTI data. To accomplish that, the deskulled FLAIR image volume was registered to the corrected b = 0 sec/mm^2^ image volume using rigid-body registration (FLIRT, FMRIB, University of Oxford, UK) [34]. The resulting transformation was then applied to the WMH mask of the participant converting it to the space of the processed DTI data.

### Association of Diffusion Measures with Systemic Inflammation: Voxel-Wise Investigation with Tract-Based Spatial Statistics

The Tract-Based Spatial Statistics (TBSS) approach was used to investigate the association of white matter diffusion measures with systemic inflammation [39]. The FA volumes from all participants were non-linearly spatially transformed to the mean FA space of the IIT Human Brain Atlas (v.3) (www.iit.edu/~mri) [40], [41]. The local FA maxima from each participant's spatially transformed FA volume were then projected onto the white matter skeleton of the IIT Human Brain Atlas (v.3) (this is produced by thinning the mean FA template of the atlas; see Reference 39). The same projection parameters were used to project the trace, axial diffusivity, radial diffusivity, and WMH mask values from the same voxels as the local FA maxima. Linear regression was then used to test the association of FA along the white matter skeleton with systemic inflammation, controlling for age, sex, level of education, and presence of WMHs. Separate linear regression models were used to test the association of trace, axial and radial diffusivity along the white matter skeleton with systemic inflammation, controlling for the same factors mentioned above. The null distribution was built using the “randomise” tool in FSL (FMRIB, University of Oxford, UK) and 5000 permutations of the data. Differences were considered significant at p<0.05, Family Wise Error (FWE) corrected. The Threshold-Free Cluster Enhancement (TFCE) method was used to define clusters with significant differences [42].

Associations of the composite measure of systemic inflammation with the clinical variables listed in Table 1 were investigated using Pearson's correlation and Student's t test. The TBSS analyses described above were then repeated, including as additional covariates the clinical variables with significant associations with the inflammation measure (p<0.05).

### Connectivity of Regions Showing Significant Association of Diffusion Measures with Systemic Inflammation

The connectivity of regions showing a significant association of diffusion measures with systemic inflammation was investigated. For that purpose, the cluster/s that showed a significant association of diffusion measures with systemic inflammation in the voxel-wise TBSS analysis were selected as regions of interest (ROIs). Since white matter fiber tractography using high angular resolution diffusion imaging (HARDI) data provides far superior results than that based on DTI data [43], the selected ROIs (already located in the space of the IIT Human Brain Atlas (v.3)) were used as seed/s for probabilistic fiber tracking in the HARDI template of the IIT Human Brain Atlas (v.3) [40], [41]. Because the ROIs were defined on the white matter skeleton of the atlas, they were first manually expanded to cover the corresponding white matter structures. The MRtrix toolbox [44] was then used to map 10^4^ fibers from each voxel of the expanded ROIs, with tracking step size  = 0.2 mm, radius of curvature  = 1 mm, minimum tract length  = 10 mm. Finally, track-density maps [45] were generated by mapping the number of fibers penetrating each voxel of the IIT Human Brain Atlas (v.3) and overlaid on the corresponding mean T1-weighted template [41].

### Association of Cognitive Measures with Systemic Inflammation, and the Role of Diffusion Anisotropy in this Relation

Linear regression was used to test the association of performance in each of the five cognitive domains, as well as global cognition, separately, with the composite measure of systemic inflammation, controlling for age, sex, level of education, and the clinical variables with significant associations with inflammation. The role of white matter diffusion anisotropy in this relation was investigated next. The mean FA in the ROIs that showed a significant association of diffusion measures with systemic inflammation in the voxel-wise TBSS analysis, was extracted for each subject. Linear regression was then used to test the association of performance in each of the five cognitive domains, as well as global cognition, separately, with mean FA in the selected ROIs, controlling for age, sex, level of education, and the clinical variables with significant associations with inflammation. Finally, for these cognitive domains that showed a significant link to both inflammation and mean FA from the selected ROIs, linear regression was repeated, including as independent variables in the same model both the composite measure of systemic inflammation and the mean FA, along with the covariates listed above.

Further investigation tested whether persons with MCI were driving any of the results. To accomplish that, all analyses described above were repeated after excluding persons who were diagnosed with MCI.

## Results

**Figure 1:**
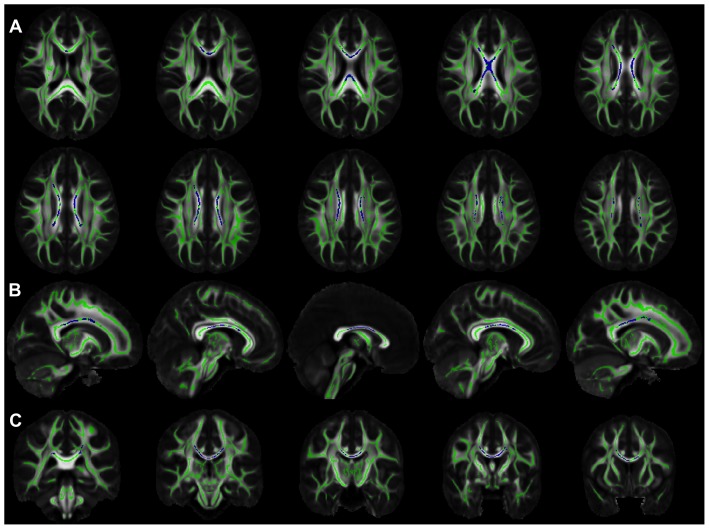
Regions of the white matter skeleton with a significant negative correlation between FA and the composite measure of systemic inflammation are shown in dark blue (controlling for age, sex, level of education, and presence of WMHs). (A) Axial (radiological convention), (B) sagittal (left to right) and (C) coronal (posterior to anterior) views are presented for better localization. In order to ensure high contrast, the same dark blue color is assigned to all voxels with p<0.05 after correction for multiple comparisons. Mean FA maps of the IIT Human Brain Atlas (v.3) (grayscale), and the corresponding white matter skeleton (green color) are shown in the background. No part of the white matter skeleton showed a significant positive correlation between FA and the composite measure of inflammation.

**Figure 2:**
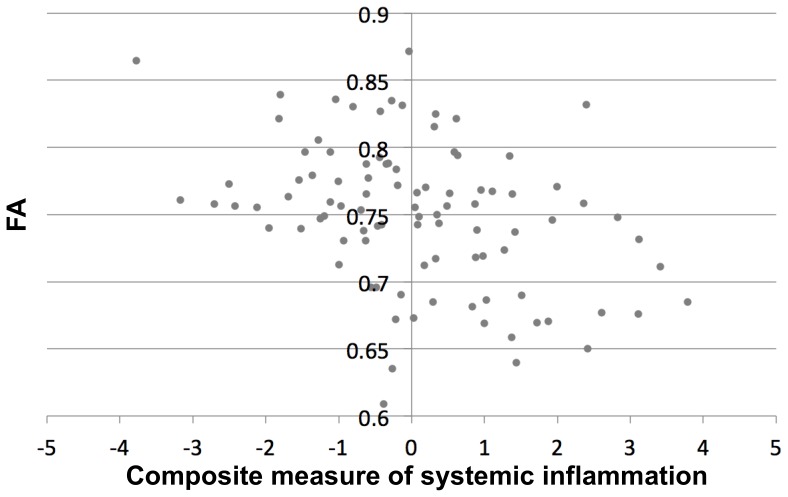
Plot of the mean FA (after correction for the effects of age, sex, level of education, and presence of WMHs) of the corpus callosum voxels that showed a significant negative correlation between FA and systemic inflammation in voxel-wise analysis (Fig. 1), as a function of the composite measure of systemic inflammation.

Demographic and clinical characteristics of the participants are presented in Table 1. TBSS analysis demonstrated significant negative correlation between FA values in the body and isthmus of the corpus callosum and the composite measure of systemic inflammation, controlling for age, sex, level of education, and presence of WMHs (p<0.05 corrected for multiple comparisons) (Fig. 1). The mean of the adjusted FA values from these voxels of the corpus callosum was plotted as a function of the composite measure of systemic inflammation (Fig. 2). No other parts of the brain showed significant negative or positive correlations between FA and systemic inflammation. Furthermore, no significant associations were detected between trace, axial or radial diffusivity and the composite measure of systemic inflammation.

Systemic inflammation was shown to be significantly higher for participants with a history of hypertension (p = 0.006) and those using antihypertensive medication at evaluation (p = 0.0003). Therefore, the second set of TBSS analyses on the link between diffusion measures and systemic inflammation controlled for age, sex, level of education, presence of WMHs, history of hypertension, and use of antihypertensive medication at evaluation. These analyses also showed significant negative correlation between FA values in the body and isthmus of the corpus callosum and the composite measure of systemic inflammation (p<0.05 corrected for multiple comparisons), in a very similar spatial pattern as the first set of TBSS analyses (Fig. 1). No parts of the brain showed significant positive correlations between FA and systemic inflammation. Finally, no significant associations were detected between trace, axial or radial diffusivity and the composite measure of systemic inflammation.

**Figure 3:**
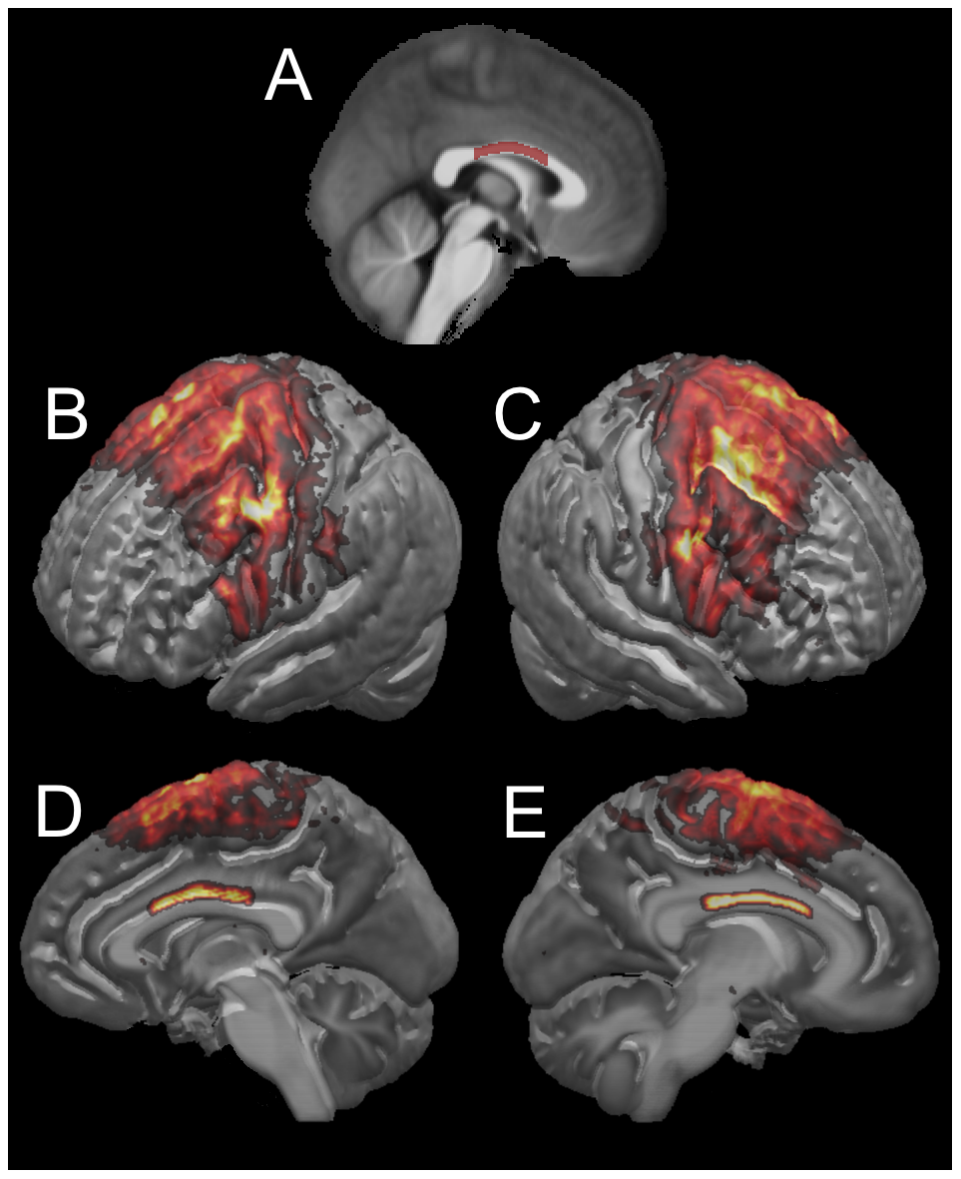
Results of probabilistic tractography in the HARDI template of the IIT Human Brain Atlas (v.3) for a seed region covering the mid-sagittal section of the corpus callosum cluster that showed significant negative correlation between FA and systemic inflammation. (A) Location of the seed used for probabilistic tractography, overlaid on the mid-sagittal slice of the mean T_1_-weighted template of the atlas. (B–E) Three-dimensional renderings of track-density maps of fibers that cross through the body and isthmus of the corpus callosum, where FA was shown to be significantly negatively correlated with the composite measure of systemic inflammation. (B) View of the lateral side of the left hemisphere. (C) View of the lateral side of the right hemisphere. (D) View of the medial aspect of the right hemisphere. (E) View of the medial aspect of the left hemisphere.

Probabilistic tractography was conducted for a seed region covering the mid-sagittal section of the corpus callosum cluster that showed significant negative correlation between FA and systemic inflammation (Fig. 3A). Track-density maps thresholded at 2000 fibers per voxel demonstrated that the seeded section of the corpus callosum contains axons connecting the following regions of the dorsal prefrontal cortex, sensory-motor cortex, and cingulate cortex: superior frontal gyrus and sulcus, middle frontal gyrus, inferior frontal gyrus and sulcus, paracentral gyrus, postcentral gyrus and sulcus, central sulcus, precentral gyrus and sulcus, cingulate gyrus and sulcus (Fig. 3B–E).

In linear regressions investigating separately the association of cognitive performance with systemic inflammation and mean FA in the cluster resulting from the voxel-wise TBSS analysis, semantic memory was significantly positively correlated with mean FA (Table 2). Additionally, visuospatial ability was significantly negatively correlated with the composite measure of systemic inflammation, and positively correlated with mean FA in the corpus callosum cluster (Table 2). When investigating the associations of visuospatial ability with both systemic inflammation and mean FA in the same linear regression model, the association with systemic inflammation became non-significant, while the association with mean FA remained significant (Table 2).

**Table 2:** Association of cognitive performance with systemic inflammation and mean FA from the region of the corpus callosum that showed significant negative correlation of FA with inflammation in the voxel-wise TBSS analysis.

	Inflammation + Covariates			FA+ Covariates			Inflammation +FA + Covariates					
	Inflammation			FA			Inflammation			FA		
	β	SE	p	β	SE	p	β	SE	p	β	SE	p
**Episodic memory**	0.03	0.05	0.48	−0.05	1.18	0.97						
**Semantic memory**	−0.06	0.04	0.16	**2.3**	**1.0**	0.03*						
**Working memory**	0.05	0.05	0.30	−0.4	1.3	0.75						
**Perceptual speed**	0.006	0.046	0.89	1.1	1.2	0.35						
**Visuospatial ability**	**−0.09**	**0.05**	0.04*	**3.1**	**1.2**	0.009*	**−0.07**	**0.05**	**0.16**	**2.6**	**1.2**	0.03*
**Global cognition**	0.002	0.032	0.94	0.8	0.8	0.3						

The results from three types of linear regression models are shown. In the first two columns, the composite measure of systemic inflammation and mean FA are included in models separately. In the last column, the two measures are included in the same model. All linear regression models contain as covariates age, sex, level of education, history of hypertension, and use of antihypertensive medication at evaluation. The estimate (β), standard error (SE) and p-value (p) are reported for each case. Significant associations are in bold letters and the p-value is marked with a *.

Of the 95 non-demented elderly participants, 11 were diagnosed with MCI. After removing all persons with MCI and repeating all analyses, the results were rather stable, with minor changes in the maps derived from the voxel-wise analyses, as well as in the estimates and p-values of all models.

## Discussion

MRI has demonstrated macro-structural brain abnormalities linked to systemic inflammation in non-demented elderly subjects. Reduced microstructural integrity in white matter has also been linked to systemic inflammation, but these investigations have focused on non-demented middle-aged adults, or a combination of middle-aged and elderly adults mostly younger than 65 years of age. The purpose of this study was to test the hypothesis that higher levels of systemic inflammation in a community sample of non-demented subjects older than 70 years of age are associated with reduced diffusion anisotropy in brain white matter and lower cognition. It was demonstrated that FA values in the body and isthmus of the corpus callosum were negatively correlated with a composite measure of systemic inflammation. Furthermore, visuospatial ability was negatively correlated with systemic inflammation, and diffusion anisotropy in the body and isthmus of the corpus callosum was shown to mediate this association. All results were robust to the exclusion of persons with MCI.

FA represents the degree of diffusion anisotropy, and, in white matter, it is dependent on microstructural tissue properties such as axonal density, degree of myelination, axonal diameter, inter-axonal spacing, and intravoxel coherence of axonal orientation [46]. The negative association of callosal FA with the composite measure of systemic inflammation in both sets of TBSS analyses suggests that the condition of one or more of these microstructural features of the corpus callosum may be poorer for higher levels of inflammation (e.g. lower axonal density, less myelin). Lower white matter FA with higher levels of inflammation has previously been translated as lower microstructural integrity of white matter [26], [27]. The link between white matter microstructure and inflammation is further supported by accumulating evidence that high inflammation levels may establish an environment that leads to, or enhances, neurodegeneration [3], [47]. Trace, axial and radial diffusivity can often provide additional (not conclusive) information on the mechanisms underlying FA changes, but no significant associations of these three parameters with inflammation were detected here, nor have previously been reported. Histological investigation of brain microstructure as a function of systemic inflammation is warranted.

Our finding of a negative correlation between FA in white matter and systemic inflammation is in general agreement with previous research [26], [27]. Conventional voxel-wise analysis of FA values in relation to CRP levels in a large cohort of middle-aged and elderly subjects mostly younger than 65 years of age, showed lower FA for higher CRP levels in the body and anterior section of the corpus callosum [26]. TBSS analysis in a cohort of middle-aged subjects revealed a significant negative correlation between FA values throughout the corpus callosum, as well as in other white matter regions throughout the brain [27]. Factors that may have led to any discrepancies in location of the findings across studies include: differences in age or other demographic characteristics, inclusion/exclusion criteria, imaging parameters, post-processing approach, the use of a composite measure of systemic inflammation (in the present study) instead of only CRP levels, and controlling for the presence of WMHs in each voxel separately (in the present study) instead of using in each voxel the same semi-quantitative severity score derived from the whole brain.

The corpus callosum is the largest white matter fiber bundle connecting the two cerebral hemispheres [48]. The body and isthmus of the corpus callosum contain mainly large diameter, fast-conducting, highly myelinated axons. Maps of the brain regions connected with callosal fibers have been constructed by means of histology [48] or, more recently, by fiber tractography based on DTI [49]–[51] or high angular resolution diffusion imaging [52]. There is excellent agreement between the present and previous studies regarding the cortical regions with interhemispheric connections through the body and isthmus of the corpus callosum (Fig. 3) [48]–[52]. Interestingly, a recent investigation of brain macrostructure in non-demented elderly persons with high levels of systemic inflammation detected cortical thinning in superior frontal, precentral, and postcentral areas (interhemispheric connections through the body and isthmus of the corpus callosum) among other regions [18]. Furthermore, an investigation of age-associated cortical thinning in 883 non-demented subjects 18–93 years of age from 6 cohorts demonstrated that the superior, middle, and inferior frontal gyri (interhemispheric connections through the body of the corpus callosum) are among the brain regions with the strongest association between cortical thinning and age [53]. The overlap in findings from the present work and previously published macrostructural studies suggests signs of “inflammaging”, the concept that common age-related pathologies and brain abnormalities may be due to low grade chronic upregulation of certain inflammatory responses associated with aging [54]. A multi-modal imaging, clinical, pathologic investigation is necessary to fully characterize the role of inflammation in brain aging.

Several studies have shown that higher levels of circulating inflammatory markers are associated with lower cognitive function and cognitive decline in aging [8], [55]–[63]. The association of systemic inflammation and domain-specific cognitive function, however, is less clear. For example, although associations with executive function [26], [55], [58], [63] and memory [55], [56], [58] are often reported, these findings are inconsistent across studies and there are reports of significant correlations between systemic inflammation and other cognitive functions such as processing speed [59], nonverbal intelligence [64] and visuospatial function [56]. In this study, systemic inflammation was negatively correlated with visuospatial ability, and diffusion anisotropy in the body and isthmus of the corpus callosum mediated this association. The corpus callosum is known to be critical for general intellectual function, with correlations reported to be strongest in the posterior body and isthmus regions [65]. The tests comprising the visuospatial domain used in this work are well-established measures of perceptual concept formation (nonverbal intelligence) and spatial cognition [66], and functional MRI studies on healthy young persons performing these particular tasks reported activation in several fronto-parietal gray matter regions known to have connections through the body and isthmus of the corpus callosum [67], [68]. Fronto-parietal volumes have been shown to be negatively associated with systemic inflammation in older persons without dementia [18] and reduced higher-order visuospatial cognition is not an uncommon finding in aging studies [69], [70]. Taken together, the findings suggest that in older persons without dementia, systemic inflammation may compromise mid-callosal tracts, with secondary effects on fronto-parietal gray matter regions that are critical for higher-order visuospatial cognition.

In conclusion, the present study demonstrated that, FA in the body and isthmus of the corpus callosum in community-dwelling non-demented individuals aged 73–100 years (85±6 years) was negatively correlated with a composite measure of systemic inflammation. These results are in general agreement with previous research on younger subjects, and may indicate lower microstructural integrity of white matter in the corpus callosum for higher levels of systemic inflammation. Visuospatial ability was negatively correlated with systemic inflammation, and FA in the body and isthmus of the corpus callosum was shown to mediate this association. In fact, visuospatial abilities involve gray matter regions with connections through the body and isthmus of the corpus callosum. Because FA is a rather non-specific measure of underlying tissue characteristics, histological investigation of brain microstructure as a function of systemic inflammation is warranted.
